# Specific allelic variants of SNPs in the *MDM2* and *MDMX* genes are associated with earlier tumor onset and progression in Caucasian breast cancer patients

**DOI:** 10.18632/oncotarget.26768

**Published:** 2019-03-08

**Authors:** Marcus Bauer, Eva Johanna Kantelhardt, Thorsten Stiewe, Andrea Nist, Marco Mernberger, Katharina Politt, Volker Hanf, Tilmann Lantzsch, Christoph Uleer, Susanne Peschel, Jutta John, Jörg Buchmann, Edith Weigert, Karl-Friedrich Bürrig, Claudia Wickenhauser, Christoph Thomssen, Frank Bartel, Martina Vetter

**Affiliations:** ^1^ Institute of Pathology, Martin-Luther-University Halle-Wittenberg, Halle (Saale), Germany; ^2^ Department of Gynaecology, Martin-Luther-University Halle-Wittenberg, Halle (Saale), Germany; ^3^ Institute of Medical Epidemiology, Biostatistics and Informatics, Martin-Luther-University Halle-Wittenberg, Halle (Saale), Germany; ^4^ Institute of Molecular Oncology, Universities of Giessen and Marburg Lung Center, German Center for Lung Research (DZL), Marburg, Germany; ^5^ Genomics Core Facility, Philipps-University, Universities of Giessen and Marburg Lung Center, German Center for Lung Research (DZL), Marburg, Germany; ^6^ Universities of Giessen and Marburg Lung Center, German Center for Lung Research (DZL), Marburg, Germany; ^7^ Department of Gynaecology, Hospital Fuerth, Fuerth, Germany; ^8^ Department of Gynaecology, Hospital St. Elisabeth and St. Barbara, Halle (Saale), Germany; ^9^ Onkologische Praxis Uleer, Hildesheim, Germany; ^10^ Department of Gynaecology, St. Bernward Hospital, Hildesheim, Germany; ^11^ Department of Gynaecology, Helios Hospital Hildesheim, Hildesheim, Germany; ^12^ Institute of Pathology, Hospital Martha-Maria, Halle (Saale), Germany; ^13^ Institute of Pathology, Hospital Fuerth, Fuerth, Germany; ^14^ Institute of Pathology Hildesheim, Hildesheim, Germany

**Keywords:** p53, mutation, tumor suppression, breast cancer, single nucleotide polymorphism

## Abstract

**Background:**

Genetic factors play a substantial role in breast cancer etiology. Genes encoding proteins that have key functions in the DNA damage response, such as p53 and its inhibitors MDM2 and MDMX, are most likely candidates to harbor allelic variants that influence breast cancer susceptibility. The aim of our study was to comprehensively analyze the impact of SNPs in the *TP53*, *MDM2*, and *MDMX* genes in conjunction with *TP53* mutational status regarding the onset and progression of breast cancer.

**Methods:**

In specimen from 815 breast cancer patients, five SNPs within the selected genes were analyzed: *TP53* – Arg72Pro (rs1042522), *MDM2* – SNP285 (rs2279744), SNP309 (rs117039649); *MDMX* – SNP31826 (rs1563828), and SNP34091 (rs4245739). Classification of the tumors was evaluated by histomorphology. Subtyping according hormone receptor status, HER2-status and proliferation rate enabled provision of the clinico-pathological surrogate of intrinsic subtypes.

**Results:**

The homozygous C-allele of *MDM2* SNP285 was significantly associated with a younger age-at-diagnosis of 44.2 years, in contrast to G/G- and G/C-patients (62.4, 62.7 yrs., respectively; *p* = 0.0007; log-Rank-test). In contrast, there was no difference regarding the age-at-diagnosis for patients with the respective genotypes of *MDM2* SNP309 (*p* = 0.799; log-Rank-test). In patients with estrogen receptor (ER)-positive and *TP53*-mutated tumors, however, the T/T-genotype of the *MDM2* SNP309 was significantly associated with an earlier average age-at-diagnosis compared with T/G+G/G-patients (53.5 vs. 68.2 yrs; *p* = 0.002; log-Rank-test). In the triple-negative subgroup, the G/G-patients had an average age-at-diagnosis of 51 years compared with 63 years for SNP309T carriers (*p* = 0.004; log-Rank-test) indicating a susceptibility of the G/G genotype for the development of triple negative breast cancer. Patients with the A/A-genotype of *MDMX* SNP31826 with ER-negative tumors were diagnosed 11 years earlier compared with patients and ER-positive tumors (53.2 vs. 64.4 yrs; *p* = 0.025, log-Rank-test). Furthermore, in luminal B-like patients (HER2-independent) the C/C-genotype of *MDMX* SNP34091 was significantly correlated with a decreased event-free survival compared with the A/A-genotype (*p* < 0.001; log-Rank-test).

**Conclusions:**

We showed that SNPs in the *MDM2* and *MDMX* genes affect at least in part the onset and progression of breast cancer dependent on the ER-status. Our findings provide further evidence for the distinct etiological pathways in ER-negative and ER-positive breast cancers.

## INTRODUCTION

Breast cancer of the most common malignancy among women in Europe and worldwide. Genetic factors play a substantial role in breast cancer etiology [1]. Specific inherited mutations in *BRCA1* and *BRCA2* increase the risk of female breast cancer. Together, *BRCA1* and *BRCA2* mutations account for about 5 to 10 percent of all breast cancers [2]. Individuals with *BRCA1* mutations are predominantly predisposed to estrogen receptor (ER)-negative breast cancer, whereas other known susceptibility loci for breast cancer are stronger associated with ER-positive tumors [1]. Patients with ER-negative tumors have a worse short-term prognosis [3] and a weaker association with reproductive risk factors [4]. Furthermore, Li-Fraumeni patients with germline mutations of *TP53* have an increased risk of developing breast cancer.

It is important to clarify the molecular mechanism of breast cancer development which can help to detect breast cancer at an early stage, and to study single nucleotide polymorphisms that affect the pathways which could be relevant for tumor formation and/or progression. Numerous studies with large patient cohorts such as the Breast Cancer Association Consortium (BCAC) have identified single nucleotide polymorphisms that are associated with the onset of breast cancer [5–8]. Genes that encode proteins with functions in the DNA damage response, such as *TP53* and its key inhibitors *MDM2* and *MDMX* [9], are most likely candidates to harbor allelic variants that influence breast cancer susceptibility.

The p53-pathway is essential for the cells’ adequate response to stress. In detail, p53 regulates transcriptional programs important in suppressing tumor formation and progression as well as the cellular response to certain therapies by regulating cell-cycle-arrest, cell death, metabolic processes, DNA repair, and others [10]. MDM2 and MDMX have indispensable roles in regulating the activity and the levels of p53 in both unstressed cells and following genotoxic stress [11]. By binding to p53, both MDM2 and MDMX inhibit p53's function as a transcription factor. Furthermore, MDM2 also mediates the proteasomal degradation of p53 [12] by serving as an E3 ligase [13]. On the other hand, p53 mediates transcription of the *MDM2* and *MDMX* gene through p53-sensitive promoters thereby forming a fine-tuned negative-feedback loop [14–16].

Several aspects of the p53-MDM2-MDMX-axis are particularly relevant to human cancer. The *TP53* gene is mutated in nearly half of all sporadic human cancers; however, there are great differences among tumor types [17]. Importantly, tumors expressing wild-type p53 are often characterized by overexpression of MDM2 or MDMX, due to gene amplification or other mechanisms, particularly in breast cancer [18, 19]. Varying transcription levels, and the subsequent biological outcome, can be explained by the impact of different alleles of single nucleotide polymorphisms (SNPs) in the *MDM2* and *MDMX* gene. In this context, Bond *et al.* have identified a SNP in the p53-sensitive P2-promoter of the *MDM2* gene – designated as SNP309 – which affects the risk of developing cancer [20] in a gender-specific and hormone-dependent manner [21]. Subsequently, however, conflicting results have been published regarding the association between SNP309G and increased cancer-risk, specifically in Caucasian populations. For example, Knappskog *et al.* could not reproduce these findings in the context of breast and ovarian cancer development [22]. Interestingly, the group described a second SNP 24 bp upstream of SNP309 in the *MDM2* P2 promoter (SNP285) and showed that the increased transcription caused by the G-allele of SNP309 is reduced by the C-allele of SNP285, whereby SNP285C and SNP309G are inherited as haplotype. Therefore, the risk of breast and ovarian cancer is decreased in SNP285C/SNP309G-haplotype carriers [22].

Similarly to MDM2, MDMX is often amplified and overexpressed in tumors (reviewed in [23]). As described for MDM2, specific SNPs in the *MDMX* gene may affect the activity of p53 and the tumor-risk [24]. Recently, we identified a SNP (SNP34091, rs4245739) in the 3′-UTR of the *MDMX* gene. The C-allele of rs4245739 creates a binding site for hsa-miR-191 and hsa-miR-887 which causes downregulation of the MDMX expression in the respective tissue [25]. In ovarian cancer, the A/A-genotype is associated with a significantly shortened disease-free and overall survival dependent on the ER-status of the tumor [25]. Several genome-wide association studies (GWAS) revealed that the 1q32-locus, including SNP34091 (rs4245739), is a susceptibility locus in ER-negative breast cancer [26] and triple-negative breast cancer [27, 28].

In the study presented here, we assessed the impact of SNPs in the *TP53*, *MDM2*, and the *MDMX* gene on the age-at-diagnosis and event-free survival in a prospective cohort of German breast cancer patients (*n* = 815) and correlated these data with (i) the *TP53*-mutational status, (ii) the hormone receptor and HER2 status as surrogate markers for the intrinsic subtypes as well as (iii) clinic-pathological data. Our data clearly indicate allele-specific effects of SNPs in critical regulators of p53 on the age-at-diagnosis as well as the event-free survival, depending on the ER-status, respectively on the intrinsic subtypes as well as the *TP53*-mutational status. Our findings provide evidence for the distinct etiological pathways in invasive ER-negative and ER-positive breast cancers.

## RESULTS

### *TP53* mutational status

**Figure 1:**
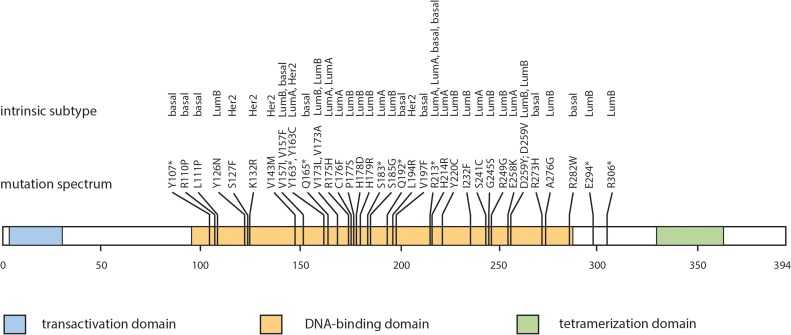
*TP53* mutation spectrum according to intrinsic subtype Mutation plot of somatic TP53 mutations (missense and nonsense mutations) in breast cancer. Abbr.: R110P – wild-type amino acid, codon, altered amino acid; Y107^*^ - wild-type amino acid, altered codon, STOP; Bas – triple-negative (ductal); Her – HER2-positive (non-luminal); LuA – luminal A-like; LuB – luminal B-like.

In a subset of 257 patients from the cohort the *TP53* mutational status was determined by NGS. Here, in 16.7% of the patients (43/257) mutations in the *TP53* were detected. All mutations were located in exons 4 through 8 and affected the amino acid sequence of p53. 79.1% (34/43) were missense mutations and 20.9% (9/43) were nonsense mutations which a single nucleotide exchange leading to a premature stop codon (Table 1, Figure 1). According to the *TP53* mutations database [29] all mutations that were detected in our cohort have already been described in the context of breast cancer.

**Table 1:** Summary of *TP53* mutations in breast cancer

Case	Histology	Exon	Codon	Conserved region	Mutation Type	Change	wt	mt	wt-AA	mt-AA
#188	basal	4	107	NC	Nonsense	C>G	TAC	TAG	Tyr	STOP
#229	basal	4	110	NC	Missense	G>C	CGT	CCT	Arg	Pro
#161	basal	4	111	NC	Missense	T>C	CTG	CCG	Leu	Pro
#277	Lum B	5	126	NC	Missense	T>A	TAC	AAC	Tyr	Asn
#287	HER2	5	127	Co	Missense	C>T	TCC	TTC	Ser	Phe
#158	HER2	5	132	Co	Missense	A>G	AAG	AGG	Lys	Arg
#225	HER2	5	143	Co	Missense	G>A	GTG	ATG	Val	Met
#396	Lum B	5	157	Co	Missense	G>A	GTC	ATC	Val	Ile
#306	basal	5	157	Co	Missense	G>T	GTC	TTC	Val	Phe
#361	Lum A	5	163	Co	Nonsense	C>A	TAC	TAA	Tyr	STOP
#516	HER2	5	163	Co	Missense	A>G	TAC	TGC	Tyr	Cys
#146	basal	5	165	NC	Nonsense	C>T	CAG	TAG	Gln	STOP
#339	Lum B	5	173	Co	Missense	G>T	GTG	TTG	Val	Leu
#209	Lum B	5	173	Co	Missense	T>C	TGT	GCG	Val	Ala
#226	Lum A	5	175	Co	Missense	G>A	CGC	CAC	Arg	His
#224	Lum A	5	175	Co	Missense	G>A	CGC	CAC	Arg	His
#404	Lum A	5	176	Co	Missense	G>T	TGC	TTC	Cys	Phe
#344	Lum B	5	177	Co	Missense	C>T	CCC	TCC	Pro	Ser
#208	Lum B	5	178	Co	Missense	C>A	CAC	AAC	His	Asn
#368	Lum B	5	179	Co	Missense	A>G	CAT	CGT	His	Arg
#425	Lum A	5	183	NC	Nonsense	C>G	TCA	TGA	Ser	STOP
#528	Lum B	5	185	NC	Missense	A>G	AGC	GGC	Ser	Gly
#451	basal	6	192	NC	Nonsense	C>T	CAG	TAG	Gln	STOP
#297	HER2	6	194	Co	Missense	T>G	CTT	CGT	Leu	Arg
#267	basal	6	197	NC	Missense	T>A	GTG	GAG	Val	Glu
#345	Lum A	6	213	Co	Nonsense	C>T	CGA	TGA	Arg	STOP
#309	basal	6	213	Co	Nonsense	C>T	CGA	TGA	Arg	STOP
#174	Lum A	6	213	Co	Nonsense	C>T	CGA	TGA	Arg	STOP
#541	basal	6	213	Co	Nonsense	C>T	CGA	TGA	Arg	STOP
#408	Lum A	6	214	Co	Missense	A>G	CAT	CGT	His	Arg
#268	Lum B	6	220	NC	Missense	A>G	TAT	TGT	Tyr	Cys
#480	Lum B	7	232	NC	Missense	A>T	ATC	TTC	Ile	Phe
#220	Lum A	7	241	Co	Missense	C>G	TCC	TGC	Ser	Cys
#228	Lum B	7	245	Co	Missense	G>A	GGC	AGC	Gly	Ser
#148	Lum B	7	249	Co	Missense	A>G	AGG	GGG	Arg	Gly
#450	Lum A	7	258	NC	Missense	G>A	GAA	AAA	Glu	Lys
#159	Lum B	7	259	NC	Missense	G>T	GAC	TAC	Asp	Tyr
#360	Lum B	7	259	NC	Missense	A>T	GAC	GTC	Asp	Val
#302	basal	8	273	NC	Missense	G>A	CGT	CAT	Arg	His
#344	Lum B	8	276	Co	Missense	C>G	GCC	GGC	Ala	Gly
#332	basal	8	282	Co	Missense	C>T	CGG	TGG	Arg	Trp
#316	Lum B	8	294	NC	Nonsense	del			FS	
#383	Lum B	8	306	NC	Nonsense	del			FS	

Abbreviations: basal – triple-negative (ductal); HER2 – HER2-positive (non-luminal); Lum A – luminal A-like; Lum B – luminal B-like (HER2-independent); NC – non-conserved region of the TP53 gene; Co – highly conserved region of the *TP53* gene; FS – frameshift; del – deletion.

Depending on the intrinsic cancer subtype frequency of *TP53* mutations varied from 11.9% (9 out of 84) in Luminal A-like tumors, 20% (17 out of 85) in Luminal B-like (HER2-independent), 37.9% (11 out of 29) in triple-negative tumors and 55.5% (5 out of 9) in HER2-positive (non-luminal) tumors. Statistic evaluation revealed that *TP*53 mutational status was associated with HER2-overexpession (*p* = 0.018, binary-logistic regression) and the lack of ER- and PR-expression (*p* < 0.001; *p* < 0.001, respectively; binary-logistic regression; Supplementary Table 1).

### Impact of *TP53* germline variations in *TP53* mutated and unmutated breast cancer

In our study, the percentages harboring the three different genotypes of the Arg72Pro-SNP were recorded to be: G/G 49.2% (406/815); G/C 43.8% (351/815), and C/C 7.0% (51/815). The genotype frequencies were found to be in Hardy-Weinberg-equilibrium (*p* > 0.95) and were comparable to the distribution in European individuals of the 1000 Genomes Project [30]. Correlation with the clinical data revealed that presence of the C-allele was significantly associated with higher histological grade of the primary tumor (OR = 1.615; *p* = 0.032; Supplementary Table 2). However, there was no association of the Arg72Pro-SNP with the age-at-diagnosis (*p* = 0.198; variance test).

**Figure 2:**
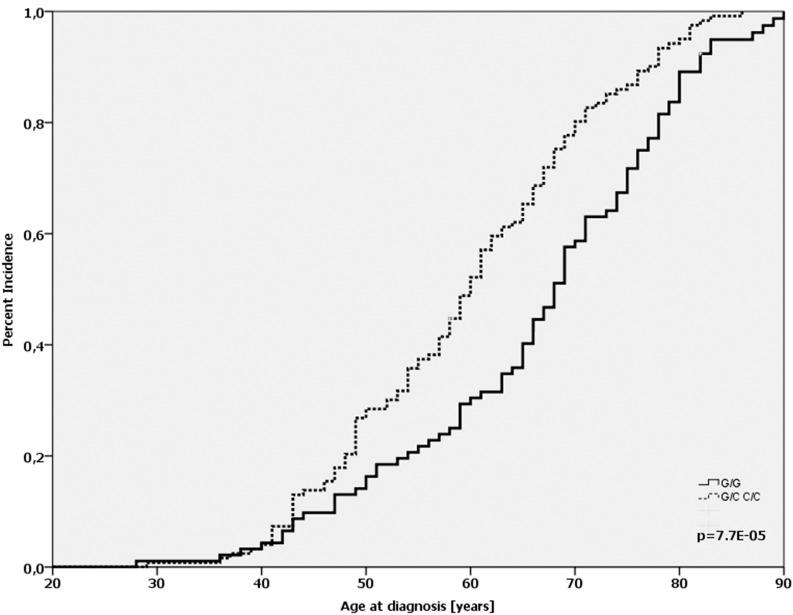
Age-at-diagnosis of the first breast cancer for patients with the different genotypes of the *TP53* Arg72Pro-SNP (G/G vs. G/C+C/C) and *TP53* wild-type gene

By separating the patients according to their *TP53* mutational status, we found that in patients whose tumors were wild-type for the *TP53* gene the homozygous Arg-allele (91/210) was significantly associated with a later age-at-diagnosis compared with patients who carried at least one Pro-allele (66.2 vs. 59.7 yrs.; *p* < 0.001; log-Rank-test; Figure 2). This effect was not observed in patients with a mutated *TP53* gene (62.3 vs. 60.3 yrs.; *p* = 0.827; log-Rank-test).

We next explored potential associations of the respective genotypes of the *TP53* Arg72Pro with differential times to recurrence and survival after diagnosis. As mentioned above, the *TP53* Arg72Pro-SNP is not associated with the age-at-diagnosis in our breast cancer patient cohort. The same applies for the impact of the respective alleles on the event-free survival (Supplementary Table 3). There was no difference regarding the time to local or distant recurrence between patients with different genotypes of the *TP53* Arg72Pro-SNP (*p* = 0.356, log-Rank-test). In addition, the *TP53* mutational status also did not affect the event-free survival time. Although patients with *TP53* wild-type tumors had an average event-free survival of 78 months compared with 70.6 months for patients with *TP53* mutated tumors, the difference was not significant (*p* = 0.311; log-Rank-test).

### *MDM2* - SNP285 and SNP309 (rs2279744 and rs117039649)

The allelic status of *MDM2* SNP309 was successfully determined in 815 cases. The T/T genotype was detected in 45.3% of the patients (369 of 815), 42.9% (350 of 815) were heterozygous (T/G), and 11.8% (96 of 815) of the patients were homozygous for the G-allele (G/G). The genotype frequencies *MDM2* SNP309 were in Hardy-Weinberg-equilibrium (*p* > 0.9) and comparable to those found in European individuals of the 1000 Genomes Project [30]. The distribution of the genotypes did not differ among tumor grades and histological subtypes. We also did not detect an association with the ER- and PR-status of the patient's tumor (*p* = 0.177, *p* = 0.74, respectively; binary-logistic regression; Supplementary Table 4).

The G-allele of SNP309 has been shown to be associated with an earlier age-at-diagnosis in numerous tumor types, including breast cancer (Review in [9]). Therefore, we compared the age when the tumor was diagnosed in the patients of our cohort according to their genotype. There was no difference regarding the age-at-diagnosis for patients with the respective genotypes of SNP309. Breast cancer patients with the T/T genotype were diagnosed at 61.9 years, T/G patients at 62.6 years, and patients who carried the homozygous G-allele at 61.6 years, respectively (*p* = 0.826, log-Rank-test; Supplementary Table 5).

Since it has been shown that SNP309 accelerates tumor formation in a gender-specific and hormone-dependent manner [21], we analyzed patients with ER-negative (*n* = 126) and ER-positive (*n* = 689) tumors separately. This revealed that the average age-at-diagnosis of patients with the T/T-T/G genotype whose tumors were ER-negative was 55.4 years compared with 62.1 years for G/G-patients (*p* = 0.091; log-Rank-test). Surprisingly, there was no difference regarding the age-at-diagnosis in patients with ER-positive tumors (62.3 vs. 62.5 yrs; *p* = 0.986; log-Rank-test). The distribution of the SNP309 genotypes was comparable between ER-negative and ER-positive tumors.

Next, we looked for an association between SNP309, the *TP53* mutational status and the age-at-diagnosis. We found that in patients with a mutated *TP53* gene, the T/T-status of SNP309 was significantly associated with an earlier age-at-diagnosis compared with T/G-G/G-patients (56.9 vs. 65.2 yrs; *p* = 0.04; log-Rank-test; Supplementary Table 5). In patients with *TP53* wild-type tumors, no difference was observed (T/T: 62 yrs., T/G-G/G: 62.8 yrs.; *p* = 0.668; log-Rank-test). A striking difference of 14.7 years regarding the age-at-diagnosis was observed in a subgroup of patients (*n* = 23) whose tumors were ER-positive and harbored a mutated *TP53* gene. Patients with a homozygous T-allele were diagnosed on average at the age of 53.5 years compared with 68.2 years for T/G-G/G-patients (*p* = 0.002; log-Rank-test).

In order to assess the potential impact of SNP309 on the luminal vs. non-luminal subtypes of breast cancer, we compared the frequency of SNP309 genotypes in the respective subgroups, which were surrogate definitions of intrinsic subtypes determined by immunohistochemistry (Table 2). We observed a slightly higher frequency of the T/T-genotype in triple-negative (ductal) tumors (54.3%) compared with luminal A-like, luminal B-like (HER2-independent), and HER2-positive (non-luminal) tumors (44.9%, 43.5%, 45.9%, respectively), though this association was not significant. We next explored, whether the genotypes of SNP309 are associated with the age-at-diagnosis within the histopathologically determined subtypes. In luminal A-like and luminal B-like (HER2-independent) breast cancer, the different alleles of SNP309 had no impact on the age-at-diagnosis (*p* = 0.966, *p* = 0.911, respectively; log-Rank-test). In the HER2-positive (non-luminal) subtype, however, the T/T-genotype of SNP309 (*n* = 17) was associated with a significantly earlier age-at-diagnosis compared with the T/G-G/G-genotypes (57.8 vs. 63.3 yrs; *p* = 0.016; log-Rank-test). In contrast, in the triple-negative (ductal) subgroup the G/G-patients (*n* = 8) had an average age-at-diagnosis of 51 years compared with 63 years for SNP309T carriers (*p* = 0.004; log-Rank-test).

**Table 2:** Surrogate definitions of intrinsic subtypes

Subtype	Characteristics
**Luminal-A-like**	HR positive HER2 negative G1, G2
**Luminal-B-like (HER2-independent)**	HR positive HER2 negative + G3 HER2 positive + all grades
**HER2 positive**	HR negative HER2 positive
**triple negative tumors**	HR negative HER2 negative

**Figure 3:**
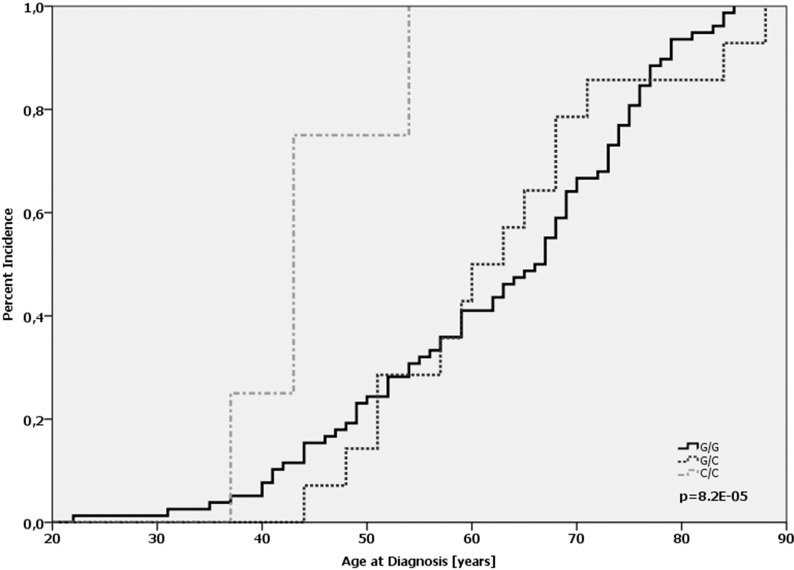
Age-at-diagnosis of the first breast cancer for patients with the different genotypes of *MDM2* SNP285 (G/G vs. G/C vs. C/C)

Furthermore, we assessed the potential impact of *MDM2* SNP285 alone or in combination with *MDM2* SNP309. Our patient cohort consisted exclusively of Caucasian women and the distribution of respective alleles matched to published frequencies observed in people cohorts from Norway and the Netherlands [22]. 92.6% (764 of 825) of the cases were homozygous for the G-allele, 6.9% (57 of 825) were heterozygous G/C, and 0.5% (4 of 825) carried the homozygous C-allele The genotype frequencies *MDM2* SNP285 were in Hardy-Weinberg-equilibrium (*p* > 0.95). The C/C genotype was associated with an increased tumor grade (OR = 1.67; *p* = 0.044; Supplementary Table 6). Comparing the age of onset revealed that patients with SNP285 C/C (*n* = 4) were diagnosed on average at the age of 44.2 (95% CI: 37.9 – 48.1), whereas the average age-at-diagnosis of G/G patients was 62.4 and 62.7 for G/C patients, respectively (*p* = 0.0007; log-Rank-test; Figure 3, Supplementary Table 7). It must be emphasized that only four patients carried the C/C genotype (see above); nonetheless, the difference is highly significant. Next, we evaluated the combination of SNP285 and SNP309 on the age of onset among breast cancer patients. The MDM2 SNP 309 G/G genotype was observed in 11.8% of patients (96 of 815) and only 4 of these showed the homozygous SNP285C/309G haplotype. Also in this subgroup SNP285 C/C dramatically reduced the age of onset only among patients with a SNP309 G/G status (44.2 years vs. 61.4 years in all patients with SNP 309 GG). There was neither a correlation of the C/C gene status with the immunohistochemically determined intrinsic subtype (*p* = 0.336; chi-square-test) nor with the ER-status of the tumor (*p* = 0.15, chi-square-test). Despite this low age-at-diagnosis, only one patient was diagnosed with recurrence after 18 months. No event was detected in the remaining C/C-patients.

The comparison of the event-free survival for patients with the different genotypes of the *MDM2* SNP309 revealed that none of the alleles was associated with the time span until the occurrence of local or distant recurrence (*p* = 0.357; log-Rank-test). In addition, we could not find any association of the alleles of SNP309 with the time to recurrence when we separated the patients according to their status of ER- (*p* = 0.487; log-Rank-test), and HER2-expression (*p* = 0.329; log-Rank-test) as well as *TP53* mutation (*p* = 0.303; log-Rank-test). Interestingly, patients with triple-negative (ductal) tumors and a homozygous T-allele of SNP309 had an event-free survival of 58.9 months compared with 66.2 months for patients with at least one G-allele, though the difference was not significant (*p* = 0.227; log-Rank-test). In contrast, the respective genotypes of SNP309 were not associated with either shortened or prolonged event-free survival within luminal A-like, luminal B-like (HER2-independent), and HER2-positive subtypes.

### *MDMX* SNP31826

The allelic status of *MDMX* SNP31826 was analyzed in 815 cases. The percentages harboring the three different genotypes were recorded to be 40.6% (331 of 815), 51.8% (422 of 815), and 7.6% (62 of 815), respectively. The genotype frequencies *MDMX* SNP31826 were in Hardy-Weinberg-equilibrium (*p* > 0.95) and comparable to those found in European individuals of the 1000 Genomes Project [30]. There were no significant associations of *MDMX* SNP31826 and clinico-pathological parameters such as tumor grading, histological subtype and hormone receptor status (Supplementary Table 8). However, patients with the homozygous A-allele were characterized by a 1.3-fold elevated risk for infiltrations of lymphatic vessels (*p* = 0.045; binary-logistic regression; Supplementary Table 8).

**Figure 4:**
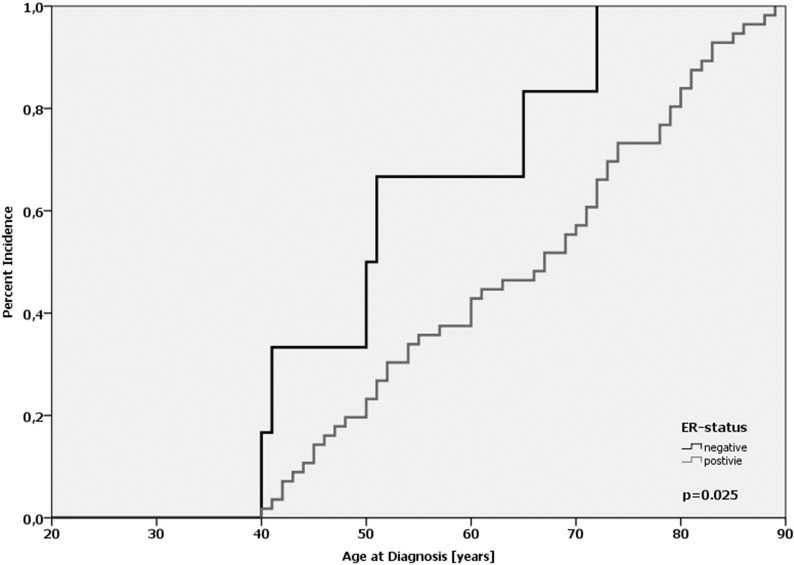
Age-at-diagnosis of the first breast cancer for patients with the A/A-genotype of *MDMX* SNP31826 and different ER-expression status

In our cohort, there was no statistically significant difference regarding the average age-at-diagnosis between the *MDMX* genotypes (*p* = 0.284; log-Rank-test; Supplementary Table 9). For the homozygous wild-type (G/G), the heterozygote (G/A) and the homozygous variant (A/A) the average age-at-diagnosis was 62.6, 61.5 and 63.3 years, respectively. In the subgroup of ER-positive tumors (*n* = 689), no difference regarding the age-at-diagnosis was observed (*p* = 0.28; log-Rank-test; Supplementary Table 9). In ER-negative tumors (*n* = 126), however, there was a left shift in the cumulative incidence curve corresponding to a 10.6 years earlier age-at-diagnosis for patients with the A/A-genotype (*n* = 6) compared with the G/G-genotype (*n* = 53); however, the difference was not significant (*p* = 0.121; log-Rank-test). Notably, the difference was 11.2 years when ER-positive and ER-negative patients with the A/A-genotype were compared (64.4 vs. 53.2 yrs; *p* = 0.025, log-Rank-test; Figure 4). Interestingly, women with the homozygous A-allele were at least 40 years old when they were diagnosed with breast cancer. Taken together, these results underline the data published by Kulkarni *et al.*, that *MDMX* SNP31826 is significantly linked to an earlier onset of disease in ER-negative breast cancer [31].

The *TP53* mutational status did not associate with a different age-of-diagnosis in patients with the respective *MDMX* SNP31826 alleles. In patients whose tumors harbor a wild-type *TP53* gene, the average age-at-diagnosis was 61.5, 63.1 and 61.3 years for the homozygous wild-type (G/G), the heterozygote (G/A) and the homozygous variant (A/A) (*p* = 0.734; log-Rank-test), and 58 and 62.3 years for the homozygous wild-type (G/G) and the heterozygote (G/A) allele in p53-mutated tumors (*p* = 0.729; log-Rank-test; Supplementary Table 9). Furthermore, we identified a subgroup of patients – albeit small – that is characterized by a notable early age-of-diagnosis. Since the risk for breast cancer is related to age at menopause, we analyzed the age-of-diagnosis in patients before the age of 55. Our data revealed that the average age-at-diagnosis of patients with ER-negative, p53-mutated tumors and the G/G-genotype of *MDMX* SNP31826 was 37 years compared with 50 years for G/A-patients (*p* = 0.001; log-Rank-test). This was not observed in patients with ER-positive tumors, *TP53*-mutated tumors (*p* = 0.998; log-Rank-test) nor in patients with *TP53*-wild-type tumors irrespective of the ER-status (data not shown).

**Figure 5:**
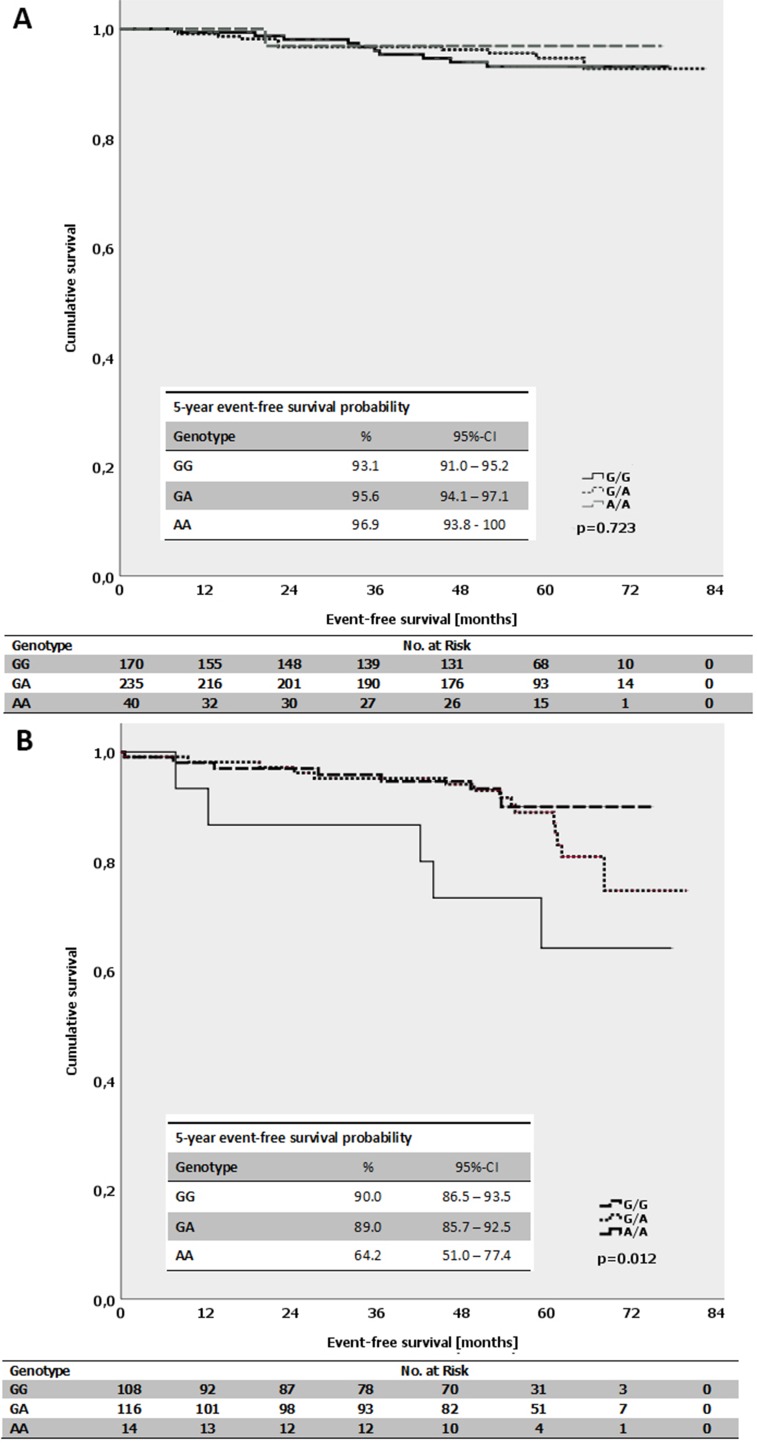
(**A**) Kaplan-Meier survival estimates for the event-free survival for patients with the different genotypes of the *MDMX* SNP31826 (G/G vs. G/A vs. A/A) in patients with luminal A-like subtype. (**B**) Kaplan-Meier survival estimates for the event-free survival for patients with the different genotypes of the *MDMX* SNP31826 (G/G vs. G/A vs. A/A) in patients with luminal B-like subtype (HER2-independent).

The average event-free survival time for patients with luminal B-like (HER2-independent) tumors and a homozygous A-allele of *MDMX* SNP31826 was 62.3 months compared with 70.6 months for patients with a homozygous G-allele (*p* = 0.012; log-Rank-test; Figure 5B). In contrast, there was no difference for patients with luminal A-like tumors and the respective genotypes (*p* = 0.723; log-Rank-test; Figure 5A). Interestingly, in triple-negative (ductal) tumors, no events occurred in the subgroup of patients with homozygous A-allele. The average event-free survival for all triple-negative (ductal) patients was 63.3 months. There was no impact of the respective MDMX SNP31826 alleles on the event-free survival within other subgroups, e.g. patients with different ER- or PR-expression status.

With respect to the influence of the respective alleles of *MDMX* SNP31826 on the age-at-diagnosis and event-free survival, there were no significant differences in our patient cohort (Supplementary Table 9).

### *MDMX* SNP34091

The allelic status of *MDMX* SNP34091 was determined in 815 breast cancer patients. The wild-type A/A-genotype accounted for 52.3% of the patients (426 of 815), 36.6% were heterozygous A/C (332 of 815), and 7% were homozygous for the C-allele (57 of 815). This is comparable to the allelic distribution found in healthy individuals (57.4%, 36.6%, 6%) [25]. The genotype frequencies *MDMX* SNP34091 were in Hardy-Weinberg-equilibrium (*p* > 0.5). The 1q32 locus where the *MDMX* gene resides has been shown to be a risk-factor of ER-negative breast cancer [26, 28]. We therefore asked whether the impact of the respective alleles of *MDMX* SNP34091 might be dependent on the ER-expression status of the tumor. We show that the A/A-genotype is associated with a 1.8-fold increased risk to develop an ER-positive tumor compared with genotypes with at least one C-allele (*p* = 0.042; binary-logistic regression, Supplementary Table 10).

*MDMX* SNP34091 A/A+A/C patients were diagnosed on average at the age of 62.2 years, whereas C/C patients at the age of 64.6 years. This difference, however, was not significant (*p* = 0.222; log-Rank-test; Supplementary Table 11). When the patients were separated according to their ER-status of the tumor, no significant impact of the respective alleles of *MDMX* SNP34091 on the age-at-diagnosis was observed (ER-negative: *p* = 0.633; ER-positive: 0.219; log-Rank-test, Supplementary Table 11). Interestingly, among pre-menopausal patients whose tumors were ER-negative the C/C-genotype was significantly associated with an earlier age-at-diagnosis compared with ER-positive patients (40 vs. 46.4 yrs; *p* = 0.001; log-Rank-test). In contrast, no difference regarding the age-at-diagnosis was observed in pre-menopausal A/A-patients (*p* = 0.325; log-Rank-test) and in post-menopausal patients (A/A: *p* = 0.762; C/C: *p* = 0.816; log-Rank-test).

**Figure 6:**
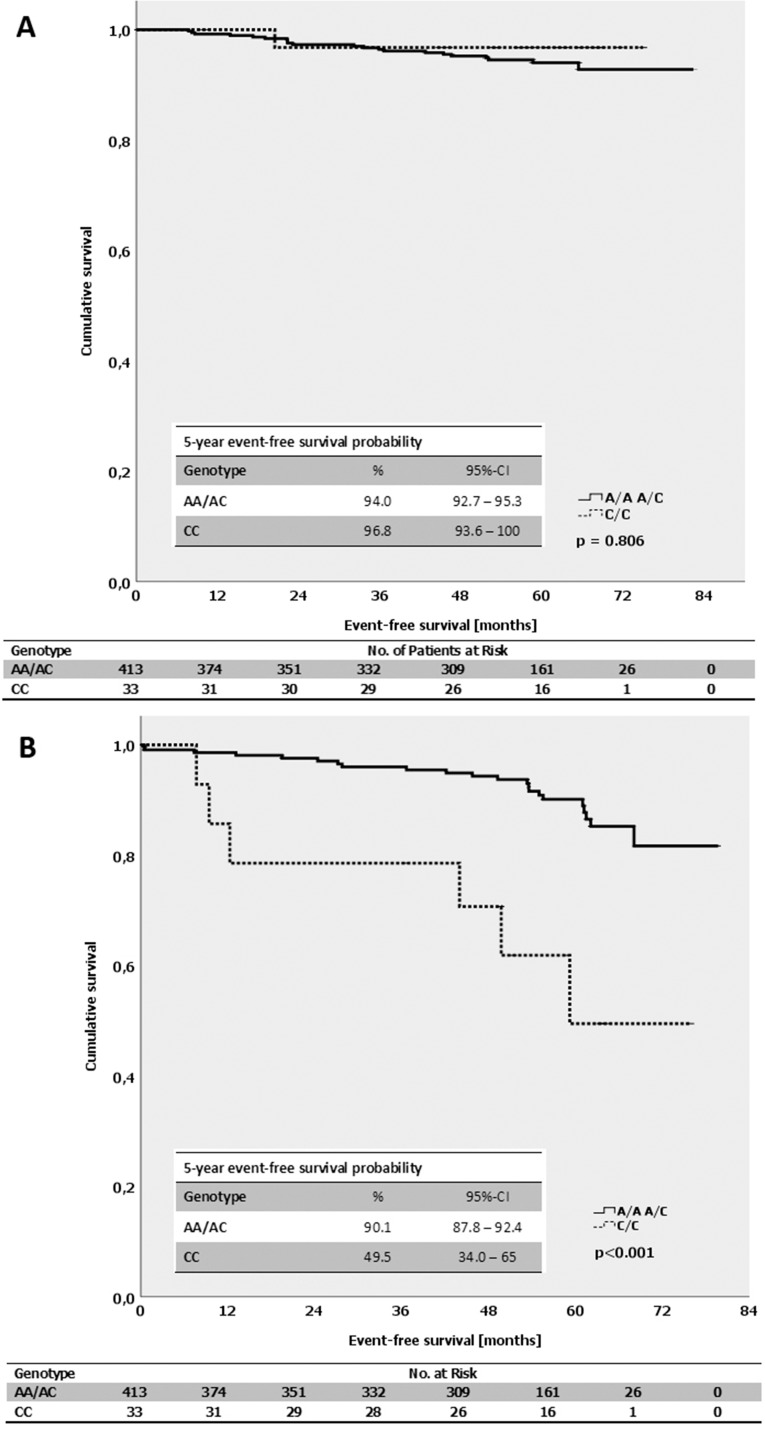
(**A**) Kaplan-Meier survival estimates for the event-free survival for patients with the different genotypes of the *MDMX* SNP34091 (A/A+A/C vs. C/C) in patients with luminal A-like subtype. (**B**) Kaplan-Meier survival estimates for the event-free survival for patients with the different genotypes of the *MDMX* SNP34091 (A/A+A/C vs. C/C) in patients with luminal B-like subtype (HER2-independent).

By evaluating the impact of the alleles of *MDMX* SNP34091 on the event-free survival in breast cancer patients, we found that A/A-patients had an average time until recurrence of 77.1 months compared with 67.3 months for C/C-patients (*p* = 0.219; log-Rank-test; Supplementary Table 11). A notable difference was observed in the luminal B-like (HER2-independent) but not in the luminal A-like and triple-negative (ductal) subtype. In luminal B-like breast cancer patients (HER2-independent), the C/C-genotype was associated with a significantly shortened event-free survival of only 54.8 months, compared with 74 months for A/A-patients (*p* < 0.001; log-Rank-test; Figure 6B). In contrast, the respective alleles of *MDMX* SNP34091 did not affect the event-free survival in patients with luminal A-like tumors (*p* = 0.806; log-Rank-test; Figure 6A). The A/A-genotype was also significantly associated with a prolonged event-free survival compared with C/C-patients in the subgroup of ER-positive (luminal) tumors (A/A: 79 months, C/C: 68 months; *p* = 0.04; log-Rank-test). However, while the event-free survival in the ER-negative subgroup (non-luminal) was shorter in general, the average time to recurrence was comparable between the A/A- and C/C-patients (A/A: 64 months, C/C: 60 months; *p* = 0.844; log-Rank-test).

To further define the influence of *MDMX* SNP34091 on age-at-diagnosis and event-free survival, we performed subgroup analysis stratified by the St. Gallen risk criteria which are briefly described in Table 3 according to Goldhirsch *et al.* [32]. Classification of patients with low, intermediate and high risk is based on clinical (age) and histopathological parameters like nodal status, hormone receptor status, and TNM [32]. Our results show that average age-at-diagnosis of patients with the C/C-genotype in the low-risk group was 51.7 years compared with 71.6 years for patients whose tumors were classified as “high risk” (*p* = 0.022; log-Rank-test; Supplementary Table 11). There was no difference regarding the age-at-diagnosis for A/A- and A/C-patients and the St. Gallen risk groups (*p* = 0.118; *p* = 0.310, respectively; log-Rank-test). Unexpectedly, the C/C-genotype in the high-risk group was associated with the shortest event-free survival time of 55.8 months compared with 68.2 months for patients in the intermediate risk-group. In addition, A/A-patients within the intermediate risk group had an event-free survival of 78.7 months, however, the difference was not significant (*p* = 0.130; log-Rank-test). No event-free survival time could be calculated for patients within the low risk-group since all cases were censored.

**Table 3:** Risk assessment according to the St. Gallen risk criteria

Risk level	Characteristics
**Low risk**	all of the following criteria: pN0, G1, pT <= 2 cm, HR-positive, V0, L0, age >= 35 years
**Intermediate risk**	pN0 and at least one of the following criteria: G2–3, T > 2 cm, HER2-positive, HR-negative, V1, age < 35 years
**High risk**	pN > 1 or pN1, HR-negative or pN1, HER2-positive

## DISCUSSION

In this descriptive, case-only study design of breast cancer patients, we examined the association of SNPs at the 1q32- and 12q13-loci and in the *TP53* gene with the onset and progression of breast cancer in a large German patient cohort with extensive clinical and pathological annotations. The study design employing solely breast cancer tissue probes was chosen, since no pathogenetic point mutations with matching exchanges in the localizations under study have been described until now. While the *TP53* Arg72Pro-SNP and the *MDM2* SNP309 have been widely studied in numerous tumor types [33], this is – to our knowledge – the first comprehensive study determining the effect of SNPs at the 1q32-locus on the onset and event-free survival in breast cancer.

Since the breast is one of the most dynamic tissues of the body, the tight control of cell proliferation during breast morphogenesis is important. Both members of the MDM family – MDM2 and MDMX – are necessary for the development and function of healthy breasts [reviewed in [34]]. The main function is dynamic negative regulation of the tumor suppressor p53. In the absence of stress, in healthy tissue, p53 levels are kept low by MDM2 and MDMX [reviewed in [35]]. In response to stress signals, however, p53 is relieved from the inhibitory effects of the MDM proteins, resulting in the accumulation of p53 and allowing it to activate appropriate pathways to ensure genomic integrity. Consistently, in normal cells of the breast duct, p53 is barely detectable while MDM2 and MDMX are at high levels [36]. It has been shown that imbalances in the expression levels of p53, MDM2 and MDMX result in the disruption of the finely tuned regulatory feedback loop. This, subsequently, might increase the risk for breast cancer [37]. Different allelic variants of the *MDM2* and *MDMX* gene have been shown to associate with altered mRNA and protein expression levels in tumors [20, 25].

The G-allele of SNP309 has been linked to early age-at-diagnosis in numerous cancer types, for example in women with soft tissue sarcomas and colorectal carcinomas [20, 21] as well as patients with ER-positive ovarian carcinomas [38]. In breast cancer, however, no association with an increased risk or earlier age-at-diagnosis was detected in a meta-analysis comprising 5,836 cases [33] and another German study [39, 40]. This is in line with the results presented here. There was no correlation of SNP309 with the age-at-diagnosis and event-free survival in our patient cohort, even after the patients were separated according to their ER-expression status. It can be concluded that the effect of the respective alleles of SNP309 is very small, and is only present in subgroups. A strength of this study is the availability of information of all study participants from pathology and clinical reports, and therefore our statistical evaluations included subtype analysis. Interestingly, the wild-type T-allele of SNP309 was associated with earlier age-at-diagnosis in ER-negative intrinsic subtypes (HER2-positive [non-luminal], triple-negative [ductal]) but not in ER-positive subtypes (luminal A-like-and luminal B-like [HER2-independent]), as well as, in patients with *TP53*-mutated but not in *TP53*-wild-type tumors. However, there was no effect of *MDM2* SNP309 on the event-free survival of the patients in none of subgroups.

The conflicting results regarding the impact of the *MDM2* SNP309 G-allele can partly be explained by a second distinct SNP in the *MDM2* P2 promoter. Knappskog *et al.* discovered a SNP, *MDM2* SNP285, which is located 24 bp upstream from *MDM2* SNP309. The C-allele of SNP285 is located on the G-allele of SNP309, forming a SNP285C/309G haplotype [22]. It has been shown that the G-allele of SNP309 increased the affinity of the transcription factor Sp1 to the MDM2 P2 promoter, leading to elevated MDM2 expression and increased cancer risk [20]. SNP285C, in contrast, significantly reduces the Sp1-binding the P2-promoter and subsequently transcription of the *MDM2* gene [22]. Consistent with this finding, individuals harboring the SNP285 C-allele had a decreased risk of breast and ovarian cancer, while the most profound effect was found in breast cancer patients with homozygous SNP309 G-allele. In contrast, in our study the C/C-genotype was associated with an extremely earlier age-at-diagnosis for patients harboring the G/G-genotype of SNP309. There was no effect of the heterozygous SNP285 C-allele (*n* = 57) on the age-at-diagnosis irrespective of the SNP309 allele-status. This is surprising since as one would expect at least slight effects of the C-allele in individuals with SNP285GC-SNP309TG+GG-genotype as it has been observed by Knappskog *et al.* [22]. On the other hand, however, the *MDM2* SNP285CC/309GG-genotype is associated with a favorable outcome, since only one patient was diagnosed with recurrence after 18 months. Interestingly, all *MDM2* SNP285 C/C patients were in the St. Gallen intermediate-risk group. Therefore, it seems reasonable to assume the SNP285 status is an additional factor for risk stratification for patients in the intermediate risk group.

Like SNP309 in the *MDM2* gene, SNPs in the *MDMX* gene are apparently correlated with early age-at-diagnosis and risk of breast cancer dependent on the ER-expression status [26, 27, 31, 41]. In our patient cohort, the homozygous A-allele of *MDMX* SNP31826 associates with an 11 years earlier age-at-diagnosis only in ER-negative breast cancers. This confirms results published by Kulkarni *et al.* [31]. In addition, the effect that the A-allele of SNP31826 is correlated with early tumor onset is restricted to the triple-negative (ductal) subtype and was not observed in patients luminal A-like or luminal B-like (HER2-independent) breast cancer. Previous data suggested that *MDM2* SNP309 is functionally active in the presence of estrogen signaling [20, 21] while the effects of *MDMX* SNP34091 are dominant in the absence of hormone signaling [25]. Recent studies, however, revealed that ERα physically interacts with both the *MDM2* and the *MDMX* gene, and that ERα expression correlates with *MDM2* and *MDMX* gene expression independent of p53 [42]. Furthermore, the authors demonstrate that expression of MDM2 and MDMX mRNA is elevated in ER-positive breast cancer samples, such as luminal A-like and luminal B-like (HER2-independent), compared with ER-negative subtypes (HER2-positive [non-luminal], triple-negative [ductal]). This suggests that effect of the *MDMX* SNPs becomes more dominant in the absence of active hormone signaling, and that subtle alterations of MDMX expression due to the different alleles may have greater impact in tumors with lack of ERα expression. Given the fact that MDM2 and MDMX have well-characterized functions in breast cancer formation and progression (reviewed in [34]), the ERα-mediated, p53-independent upregulation of MDM2 and MDMX will likely enhance these tumor-promoting processes [42]. This establishes a regulatory feedback-loop between MDM2, MDMX, and ERα. It is conceivable that specific allelic variants of MDM2 and MDMX add another layer to this fine-tuned crosstalk in breast cancer. Furthermore, these data, collectively, could explain the different effect of *MDM2* and *MDMX* SNPs in the distinct breast cancer subtypes.

Three interesting observations have been made regarding the impact of the *MDMX* SNP34091 on the age-at-diagnosis and event-free survival of breast cancer patients. Firstly, while there was no difference regarding the age-at-diagnosis of the respective genotypes for patients with ER-positive tumors, we found that C/C patients with ER-negative tumors were diagnosed either at the age of 40 or above the age of 70. Secondly, a notable difference of the event-free survival was detected only for patients with luminal B-like (HER2-independent) tumors but no other intrinsic subtypes. The C/C-genotype was associated with a significantly shortened event-free survival time of 54 months in these patients. Luminal B-like tumors are characterized by a greater percentage of *TP53* mutations, *MDM2* amplification, and higher proliferation compared to luminal A-like tumors [43]. This could, at least partly, explain the subtype-specific influence of the *MDMX* SNP34091 C/C genotype. Furthermore, C/C-patients had the shortest event-free survival time (51 months) in the St. Gallen low risk group. This is on average two years shorter than A/A-patients. The underlying mechanisms of the association of the *MDMX* SNP34091 C-allele with early tumor onset and shorter event-free survival have yet to be determined. Nonetheless, our data suggest to further elucidate its role as a risk factor in tumor prevention and as a predictor of recurrent disease.

Breast cancer is a heterogeneous disease. Attempts have been taken to define clinical and/or molecular subtypes of the disease and to elucidate driver events that are selected for during tumorigenesis. Curtis *et al.* analyzed copy number variations, SNPs and gene expression in a large cohort of breast cancer patients [44]. It has been shown that both inherited and acquired somatic alterations were associated with expression in 40% of the genes. The list of identified driver genes included both *MDM2* and *MDMX*. Furthermore, the chromosomal regions of the *MDM2* and *MDMX* gene are amplified in the majority of the integrated clusters identified by Curtis *et al.* [44]. It is conceivable that the amplification of the genes is the main reason for the overexpression of MDM2 and MDMX, and, therefore, the impact of different alleles is predominantly observable in tumors devoid of inherited or somatic copy number aberrations. High levels of MDM2 have been detected in 38% of human breast cancer cases [45]. This cannot be explained by gene amplification alone. Numerous other mechanisms contribute to increased MDM2 expression and/or activity in breast cancer, such as ERα-expression, downregulation of p14ARF, TGF1-b1-expression, as well as, expression of MDMX isoforms (reviewed in [34]).

In conclusion, we have found that specific allelic variants of the p53 tumor suppressor pathway are associated with earlier age-at-diagnosis and shortened event-free survival in subgroups of breast cancer patients. These results gained by these easily accessible and measurable biomarkers may be used in the future to identify individuals with an increased risk of developing breast cancer and to predict the responsiveness of conventional and targeted therapies. Especially, the early onset of pre-menopausal breast cancer is thought to be strongly associated with genetic predisposition. Targeted sequencing of variants in other known breast cancer susceptibility genes or genome-wide sequencing of germline variations [26, 27, 46] may provide additional clues to identify individuals with increased risk of early-onset breast cancer. Our findings need to be confirmed in independent cohorts in a subtype-specific manner and functional studies are mandatory to determine the molecular mechanisms underlying the observed effects.

## MATERIALS AND METHODS

### Patients and tissues samples

Human primary breast cancer tumors were collected at the Martin-Luther-University Halle-Wittenberg as part of the multicenter prospective PiA study (Prognostic assessment in routine Application, NCT 01592825) of unselected patients with operable and histopathological confirmed invasive breast cancer. The fresh-frozen tumor samples were derived from five different certified breast cancer centers in Germany (Martin-Luther University Halle-Wittenberg, Hospital Fuerth, Hospital St. Elisabeth & St. Barbara Halle [Saale], Breast Center Hildesheim, Breast Center Goslar [Harz]). The study was approved by the ethics committee of the Martin-Luther University Halle-Wittenberg (15.09.2009 and 10.3.2010 for patient recruitment, 15.09.2016 for this sub protocol). The PiA-study is in accordance with the Declaration of Helsinki. All patients gave their written informed consent. For tumor and patients characteristics refer to Table 4. Breast cancer subtypes were defined using histopathological information like receptor status according to the St. Gallen classification (Tables 2, 3) and by von Minckwitz and colleagues [47, 48].

**Table 4:** Summary of clinicopathologic data of patients with breast cancer

Characteristics		Patients (815)	
		No. (%)	
age at diagnosis	median		62.2 yrs.
	< 55 yrs.	270	(33.1)
	>/= 55 yrs.	545	(66.9)
T stage (primary tumor)	pT1	420	(51.5)
	pT2	349	(42.8)
	pT3	39	(4.8)
	pT4	7	(0.9)
N stage (lymph node)	N0	501	(61.5)
	N1	231	(28.3)
	N2	51	(6.3)
	N3	3	(3.9)
M stage (distant metastases)	M0	815	(100.0)
	M1	0	(0.0)
lymph vessel invasion	L0	592	(72.6)
	L1	223	(27.4)
grade	G1	94	(11.5)
	G2	508	(62.3)
	G3	211	(26.2)
histology	ductal invasive	647	(79.4)
	lobular invasive	123	(15.1)
	other	45	(5.5)
HR-status	negative	118	(14.5)
	positive	697	(85.5)
ER-status	negative	126	(15.5)
	positive	689	(84.5)
PR-status	negative	247	(30.3)
	positive	568	(69.7)
HER2-status	negative	702	(86.1)
	positive	113	(13.9)

Abbreviations: HR – hormone receptor; ER – estrogen receptor; PR – progesterone receptor; HER2 – human epidermal growth factor receptor 2.

### DNA isolation and genotyping

Genomic DNA of native tumor material was isolated using tris/triton X-100 buffer for lysis followed by centrifugation. DNA of the cell debris was isolated with the QIAamp^®^ DNA Mini Kit (Qiagen, Hilden, Germany). Five single nucleotide polymorphisms were subsequently analyzed in genes that encode proteins which are important members of the p53-pathway: p53 - rs1042522, MDM2 – rs2279744 [20], rs117039649 [22]; MDMX – rs1563828 [24], and rs4245739 [25]. To determine the allelic status of rs1042522 (p53), rs2279744 and rs117039649 (MDM2), the genomic DNA was amplified by PCR with specific primers (p53 fw: 5′-CGTTCTGGTAAGGACAAGGGT-3′, p53 rev: 5′-AAGAAATGCAGGGGGATACGG-3′; MDM2 fw: 5′-CGGGAGTTCAGGGTAAAGGT-3′; MDM2 rev: 5′-AAAGCTGAGTCAACCTGC-3′). PCR products were gel purified and subsequently analyzed by direct sequencing using the BigDye Terminator Cycle Sequencing 3.1 Kit (Applied Biosystems, Darmstadt, Germany) according to the manufacturer's intructions. The SNPs in the *MDMX* gene (rs1563828, rs4245739) were determined with a custom-made Taqman Assay by Applied Biosystems on a Rotorgene 3000 cycler (Qiagen, Hilden, Germany).

### *TP53* next generation sequencing

In a subset of 257 samples all coding exonic and flanking intronic regions of the human TP53 gene were amplified from genomic DNA with Platinum™ Taq DNA polymerase (Life Technologies) by multiplex PCR using two primer pools with 12 non-overlapping primer pairs each, yielding approximately 180 bp amplicons. Each sample was tagged with a unique 8-nucleotide barcode combination using twelve differently barcoded forward and eight differently barcoded reverse primer pools. Barcoded PCR products from up to 96 samples were pooled, purified and an indexed sequencing library was prepared using the NEBNext^®^ ChIP-Seq Library Prep Master Mix Set for Illumina in combination with NEBNext^®^ Multiplex Oligos for Illumina (New England Biolabs). The quality of sequencing libraries was verified on a Bioanalyzer DNA High Sensitivity chip (Agilent) and quantified by digital PCR. 2 × 250 bp paired-end sequencing was carried out on an Illumina MiSeq (Illumina) according to the manufacturer's recommendations at a mean coverage of 150x.

Read pairs were demultiplexed according to the forward and reverse primers and subsequently aligned using the Burrows-Wheeler Aligner against the Homo sapiens Ensembl reference (rev. 79). Overlapping mate pairs where combined and trimmed to the amplified region. Coverage for each amplicon was calculated via SAMtools (v1.1) [50]. To identify putative mutations, variant calling was performed using SAMtools in combination with VarScan2 (v2.3.9) [51]. Initially, SAMtools was used to create pileups with a base quality filter of 15. Duplicates, orphan reads, unmapped and secondary reads were excluded. Subsequently, Varscan2 was applied to screen for SNPs and InDels separately, using a low-stringency setting with minimal variant frequency of 0.1, a minimum coverage of 20 and a minimum of 10 supporting reads per variant to account for cellular and clonal heterogeneity. Minimum average quality was set to 20 and a strand filter was applied to minimize miscalls due to poor sequencing quality or amplification bias. The resulting list of putative variants was compared against the IARC TP53 (R17) database to check for known *TP53* cancer mutations.

### Statistical analyses

All statistical analyses were done by using SPSS software (version 21.0, IBM, SPSS, Inc., Chicago, IL, USA). *P* < 0.05 was determined as criterion for statistical significance for all executed statistical tests. If possible, the 95% confidence interval was calculated to declare the statistical precision of an observed effect. Furthermore, the mean age-at-diagnosis in the study cohort was examined related to the genotype. The *t*-test was used to compare two genotypes, the *F*-test to compare all three genotypes. Hazard and survival curves were generated using the Kaplan-Meier-analysis. In order to determine the event-free survival time, local, regional as well as distant recurrence, and death from breast cancer were included as “events” according to Hudis *et al.* [49]. The standardized definitions for efficacy endpoints (STEEP) were used as endpoints definitions.

## SUPPLEMENTARY MATERIALS TABLES


