# Supplementary material for: Specific allelic variants of SNPs in the MDM2 and MDMX genes are associated with earlier tumor onset and progression in Caucasian breast cancer patients
Source: Oncotarget. 2019 Mar 8;10(20):1975–92. doi: 10.18632/oncotarget.26768 (PMC6443004; doi:10.18632/oncotarget.26768)
Supplement: Supplementary file 1 [file oncotarget-10-1975-s001.pdf]

Specific allelic variants of SNPs in the *MDM2* and *MDMX* genes are associated with earlier tumor onset and progression in Caucasian breast cancer patients

SUPPLEMENTARY MATERIALS

Supplementary Table 1: Correlation of *TP53*-mutational status with clinico-pathogical parameters

variables	*TP53* mutational status	OR	95% confidence interval	P*
age at diagnosis	wt	1,000		
(< 40 yrs# vs. ≥ 40 yrs)	**mt**	**0,268**	**0,072–0,996**	**0,049**
histological subtype	wt	1,000		
(lobular# vs. NST)	mt	<0,001	-	0,998
tumor size	wt	1,000		
(T1# vs. T2-T4)	mt	0,859	0,440–1,676	0,656
nodal status (N)	wt	1,000		
(N0# vs. N1-N3)	mt	0,826	0,411–1,672	0,599
grading	wt	1,000		
(G1/2# vs. G3)	**mt**	**3,956**	**1,381–7,899**	**<0,001**
ER-status	wt	1,000		
(ER-pos.# vs. ER-neg)	**mt**	**0,147**	**0,069–0,314**	**<0,001**
PR-status	wt	1,000		
(PR-pos.# vs. PR-neg)	**mt**	**0,212**	**0,105–0,427**	**<0,001**
HER2-status	wt	1,000		
(HER2-pos.# vs. HER2-neg.)	**mt**	**2,637**	**1,181–5,890**	**0,018**
lymph vessel invasion	wt	1,000		
(V0# vs. V1)	mt	1,336	0,670–2,663	0,411
Luminal A-like-subtype	wt	1,000		
(vs. other types)	**mt**	**0,202**	**0,092–0,444**	**<0,001**
Luminal B-like-subtype	wt	1,000		
(vs. other types)	mt	1,082	0,534–2,193	0,827
HER2-positive (non-luminal) subtype	wt	1,000		
(vs. other types)	**mt**	**12,000**	**2,868–50,212**	**<0,001**
triple negative (ductal) subtype	wt	1,000		
(vs. other types)	**mt**	**0,087**	**2,069–11,002**	**<0,001**

OR: odds ratio; #reference group; *p-value from binary-logistic regressions.

Supplementary Table 2: Correlation of Arg72Pro-allele status with clinicopathogical parameters

variables	genotype	OR	95% confidence interval	p^*
age at diagnosis	G/G	1,000		
(< 40 yrs[#] vs. ≥ 40 yrs)	G/C+C/C	0,992	0,467–2,109	0,984
age at diagnosis	G/G	1,000		
(< 55 yrs[#] vs. ≥ 55 yrs)	G/C+C/C	1,034	0,772–1,384	0,823
primary tumor (T)	G/G	1,000		
(T1[#] vs. T2-T4)	G/C+C/C	1,087	0,826–1,43	0,554
nodal status (N)	G/G	1,000		
(N0[#] vs. N+)	G/C+C/C	1,232	0,929–1,634	0,147
grading	G/G	1,000		
(G1/2[#] vs. G3)	**G/C+C/C**	**1,615**	**1,041–2,505**	**0,032**
histological subtype	G/G	1,000		
(lobular[#] vs. NST)	**G/C+C/C**	**1,558**	**1,053–2,304**	**0,026**
lymph vessel invasion	G/G	1,000		
(V0[#] vs. V1)	G/C+C/C	1,023	0,752–1,392	0,886
ER-status	G/G	1,000		
(ER-pos.[#] vs. ER-neg)	G/C+C/C	0,955	0,653–1,369	0,811
PR-status	G/G	1,000		
(PR-pos.[#] vs. PR-neg)	G/C+C/C	1,049	0,778–1,414	0,755
HER2-status	G/G	1,000		
(HER2-pos.[#] vs. HER2-neg.)	G/C+C/C	1,265	0,849–1,885	0,248

OR: odds ratio; [#]reference group; [*]*p*-value from binary-logistic regressions.

Supplementary Table 3: Kaplan-Meier analysis of age-at-diagnosis and event-free survival for *TP53* Arg72Pro-SNP

		**age-at-diagnosis**		**event-free survival**	
**Variables**	**Genotype**	***years***	***P***	***months***	***P***
**all patients**	GG	62.8	0.051	73.3	0.356
	GC	61.2		76.1	
	CC	63.8		73.9	
***TP53* mutations**					
wild-type	GG	66.0	**<0,001**	--[#]	
	GC	59.4		--	
	CC	61.4		--	
mutated	GG	60.3	0.994	--[#]	
	GC	62.0		--	
	CC	56.5		--	
**ER-status**					
negative	GG	63.6	**0.05**	--[#]	--
	GC	58.7		--	
	CC	66.5		--	
positive	GG	62.6	0.211	74.8	0.663
	GC	61.8		77.6	
	CC	63.6		73.8	
**HER2-status**					
negative	GG	63.2	0.112	73.5	0.516
	GC	60.0		77.0	
	CC	66.0		73.6	
positive	GG	62.7	0.145	--[#]	--
	GC	61.4		--	
	CC	63.7		--	
**intrinsic subtypes**[*]					
Luminal A-like	GG	61.9	0.287	76.2	0.981
	GC	61.3		79.3	
	CC	64.6		73.2	
Luminal B-like	GG	64.2	0.098	--[#]	--[#]
	GC	61.7		--	
	CC	61.9		--	
HER2-positive (non-luminal)	GG	62.1	**0.028**	--[#]	--[#]
	GC	60.4		--	
	CC	*48.0*		--	
triple negative (ductal)	GG	63.8	**0.048**	--[#]	--[#]
	GC	59.9		--	
	CC	*75.0*		--	
**St. Gallen risk groups**					
low	GG	60.4	0.123	54.5	0.134
	GC	55.6		53.7	
	CC	63.1		53.0	
intermediate	GG	63.1	0.107	49.1	0.121
	GC	61.5		51.1	
	CC	64.6		48.1	
high	GG	61.6	0.917	47.7	0.700
	GC	62.5		45.3	
	CC	58.2		49.2	

[#]no calculation since all C/C-cases censored.
[*]surrogate definitions of intrinsic subtypes determined by immunohistochemistry.

Supplementary Table 4: Correlation of *MDM2* SNP309-allele status with clinicopathogical parameters

variables	genotype	OR	95% confidence interval	p^*
age at diagnosis	T/T	1,000		
(< 40 yrs# vs. ≥ 40 yrs)	T/G+G/G	0,953	0,448–2,029	0,901
age at diagnosis	T/T	1,000		
(< 55 yrs# vs. ≥ 55 yrs)	T/G+G/G	0,962	0,717–1,289	0,793
primary tumor (T)	T/T	1,000		
(T1# vs. T2-T4)	T/G+G/G	1,023	0,776–1,348	0,87
nodal status (N)	T/T	1,000		
(N0# vs. N1-N3)	T/G+G/G	0,889	0,669–1,181	0,417
grading	T/T	1,000		
(G1/2# vs. G3)	T/G+G/G	1,079	0,7–1,664	0,731
histological subtype	T/T	1,000		
(lobular# vs. NST)	T/G+G/G	0,861	0,583–1,273	0,453
lymph vessel invasion	T/T	1,000		
(V0# vs. V1)	T/G+G/G	1,052	0,772–1,433	0,748
ER-status	T/T	1,000		
(ER-pos. vs. ER-neg)	T/G+G/G	0,769	0,526–1,125	0,177
PR-status	T/T	1,000		
(PR-pos. # vs. PR-neg)	T/G+G/G	0,950	0,704–1,283	0,74
HER2-status	T/T	1,000		
(HER2-pos. # vs. HER2-neg.)	T/G+G/G	0,806	0,538–1,206	0,294

OR: odds ratio; #reference group; *p-value from binary-logistic regressions.

Supplementary Table 5: Kaplan-Meier analysis of age-at-diagnosis and event-free survival for *MDM2* SNP09

		age-at-diagnosis		event-free survival	
**Variables**	**Genotype**	***years***	***P***	***months***	***P***
**all patients**	TT	61.9	0.826	75.1	0.357
	TG	62.6		73.2	
	GG	61.6		72.8	
***TP53* mutations**					
wild-type	TT	61.7	0.300	76.4	0.701
	TG	64.0		74.5	
	GG	59.7		75.4	
mutated	TT	56.9	0.126	--[#]	
	TG	63.6		--	
	GG	67.2		--	
**ER-status**					
negative	TT	61.5	0.240	62.2	0.854
	TG	62.8		65.0	
	GG	55.5		63.3	
positive	TT	62.1	0.953	76.9	0.523
	TG	62.5		74.3	
	GG	62.5		73.7	
**HER2-status**					
negative	TT	62.6	0.655	75.3	0.078
	TG	62.8		74.1	
	GG	61.5		73.4	
positive	TT	57.3	0.059	70.0	0.463
	TG	61.2		66.2	
	GG	61.9		64.1	
**intrinsic subtypes**[*]					
Luminal A-like	TT	61.5	0.899	77.9	0.074
	TG	62.3		74.2	
	GG	61.7		67.6	
Luminal B-like	TT	62.9	0.842	73.0	0.684
	TG	62.6		71.5	
	GG	64.3		70.4	
HER2-positive (non-luminal)	TT	57.8	0.053	--[#]	
	TG	64.4		--	
	GG	61.2		--	
triple negative (ductal)	TT	63.3	**0.013**	58.9	0.399
	TG	63.7		67.5	
	GG	51.9		58.8	
**St. Gallen risk groups**					
low	TT	57.8	0.952	53.1	0.759
	TG	58.9		53.0	
	GG	58.0		63.0	
intermediate	TT	62.1	0.916	49.5	0.920
	TG	62.8		49.6	
	GG	62.9		51.3	
high	TT	62,8	0.164	43.5	0.129
	TG	62.5		49.6	
	GG	52.4		50.4	

[#]all cases censored.
[*]surrogate definitions of intrinsic subtypes determined by immunohistochemistry.

Supplementary Table 6: Correlation of *MDM2* SNP285-allele status with clinicopathogical parameters

variables	genotype	OR	95% confidence interval	p^*
age at diagnosis	G/G	1,000		
(< 55 yrs[#] vs. ≥ 55 yrs)	G/C+C/C	0,996	0,586–1,692	0,987
primary tumor (T)	G/G	1,000		
(T1[#] vs. T2-T4)	G/C+C/C	1,562	0,911–2,68	0,105
nodal status (N)	G/G	1,000		
(N0[#] vs. N1-N3)	G/C+C/C	1,513	0,855–2,679	0,155
grading	**G/G**	**1,000**		
(G1/2[#] vs. G3)	**G/C+C/C**	**1,670**	**1,014–4,813**	**0,044**
histological subtype	G/G	1,000		
(lobular vs. NST)	G/C+C/C	0,779	0,392–1,551	0,478
lymph vessel invasion	G/G	1,000		
(V0[#] vs. V1)	G/C+C/C	1,551	0,808–2,979	0,187
ER-status	G/G	1,000		
(ER-pos. [#] vs. ER-neg)	G/C+C/C	1,102	0,543–2,235	0,788
PR-status	G/G	1,000		
(PR-pos. [#] vs. PR-neg)	G/C+C/C	1,260	0,725–2,191	0,412
HER2-status	G/G	1,000		
(HER2-pos. [#] vs. HER2-neg.)	G/C+C/C	0,905	0,433–1,894	0,792

OR: odds ratio; [#]reference group; [*]*p*-value from binary-logistic regressions.

Supplementary Table 7: Kaplan-Meier analysis of age-at-diagnosis and event-free survival for *MDM2* SNP285

		age-at-diagnosis		event-free survival	
**Variables**	**Genotype**	***years***	***P***	***months***	***P***
**all patients**	GG	62.3	**<0.001**	76.6	0.542
	GC	62.5		69.9	
	CC	44.2		54.9	
***TP53* mutations**					
wild-type	GG	62.6	**<0,001**	--[#]	--
	GC	61.6		--	
	CC	43.0		--	
mutated	GG	62.0	n.d.[*]	--[#]	
	GC	54.3		--	
	CC	*37.0*		--	
**ER-status**					
negative	GG	61.3	0.074	64.8	0.488
	GC	65.9		58.7	
	CC	45.5		42.4	
positive	GG	62.4	**<0.001**	--[#]	--
	GC	62.0		--	
	CC	43.0		--	
**HER2-status**					
negative	GG	62.7	**0.004**	77.1	0.241
	GC	62.7		70.0	
	CC	46.7		49.4	
positive	GG	59.7	n.d.[*]	--[#]	--[#]
	GC	61.8		--	
	CC	*37.0*		--	
**intrinsic subtypes[+]**					
Luminal A-like	GG	62.0	**0.007**	--[#]	--[#]
	GC	61.1		--	
	CC	*43.0*		--	
Luminal B-like	GG	63.0	n.d.[*]	--[#]	--[#]
	GC	63.4		--	
	CC	43.0		--	
HER2-positive (non-luminal)	GG	n.d.[*]	n.d.[*]	--[#]	--[#]
	GC			--	
	CC			--	
triple negative (ductal)	GG	62.2	n.d.[*]	--[#]	--[#]
	GC	64.4		--	
	CC	54.0		--	
**St. Gallen risk groups**					
low	GG	58.0	0.973	54.6	0.826
	GC	62.5		37.8	
	CC	-		-	
intermediate	GG	62.7	**<0.001**	49.5	0.695
	GC	62.0		53.8	
	CC	44.3		48.3	
high	GG	61.6	0.936	46.5	0.299
	GC	66.4		51.3	
	CC	-		-	

[#]no calculation since all cases censored.
[*]n.d. – not determined, too few cases.
[+]surrogate definitions of intrinsic subtypes determined by immunohistochemistry.

Supplementary Table 8: Correlation of *MDMX* SNP31826-allele status with clinicopathogical parameters

variables	genotype	OR	95% confidence interval	p^*
age at diagnosis	G/G oder G/A	1,000		
(< 40 yrs# vs. ≥ 40 yrs)	A/A	**2,317**	**1,168–4,813**	**0,017**
age at diagnosis	G/G oder G/A	1,000		
(< 55 yrs# vs. ≥ 55 yrs)	A/A	1,125	0,885–1,43	0,337
primary tumor (T)	G/G oder G/A	1,000		
(T1# vs. T2-T4)	A/A	1,073	0,857–1,l344	0,538
nodal status (N)	G/G oder G/A	1,000		
(N0# vs. N1-N3)	A/A	1,042	0,827–1,312	0,727
grading	G/G oder G/A	1,000		
(G1/2# vs. G3)	A/A	0,937	0,66–1,33	0,716
histological subtype	G/G oder G/A	1,000		
(lobular# vs. NST)	A/A	1,129	0,826–1,543	0,447
lymph vessel invasion	G/G oder G/A	1,000		
(V0# vs. V1)	**A/A**	**1,292**	**1,005–1,66**	**0,045**
ER-status	G/G oder G/A	1,000		
(ER-pos. # vs. ER-neg)	A/A	1,148	0,838–1,571	0,391
PR-status	G/G oder G/A	1,000		
(PR-pos. # vs. PR-neg)	A/A	1,256	0,98–1,61	0,071
HER2-status	G/G oder G/A	1,000		
(HER2-pos. # vs. HER2-neg.)	A/A	0,853	0,613–1,186	0,344

OR: odds ratio; #reference group; *p-value from binary-logistic regressions.

Supplementary Table 9: Kaplan-Meier analysis of age-at-diagnosis and event-free survival for *MDMX* SNP31826

		age-at-diagnosis		event-free survival	
**Variables**	**Genotype**	***years***	***P***	***months***	***P***
**all patients**	GG	62.6	0.284	72.3	0.801
	GA	61.5		76.4	
	AA	63.3		71.4	
***TP53* mutations**					
wild-type	GG	60.9	0.734	73.7	0.858
	GA	63.1		78.0	
	AA	61.4		71.9	
mutated	GG	58.0	0.729	--[#]	
	GA	62.3		--	
	AA	-		--	
**ER-status**					
negative	GG	60.8	0.121	63.4	0.953
	GA	62.6		63.9	
	AA	53.2		58.6	
positive	GG	61.6	0.179	73.8	0.527
	GA	62.6		77.9	
	AA	64.4		71.3	
**HER2-status**					
negative	GG	62.2	0.391	72.2	0.866
	GA	62.7		77.3	
	AA	63.9		71.8	
positive	GG	57.5	0.262	69.4	0.367
	GA	61.8		65.8	
	AA	59.8		64.4	
**intrinsic subtypes**[*]					
Luminal A-like	GG	60.4	0.085	74.3	0.723
	GA	62.5		79.3	
	AA	64.3		74.6	
Luminal B-like	GG	63.3	0.523	70.6	0.058
	GA	62.3		72.7	
	AA	64.7		62.3	
HER2-positive (non-luminal)	GG	60.5	0.269	66.1	0.112
	GA	62.2		62.8	
	AA	51.0		45.0[+]	
triple negative (ductal)	GG	61.7	0.171	--[#]	
	GA	63.6		--	
	AA	53.6		--	
**St. Gallen risk groups**					
low	GG	59.2	0.404	47.1	0.723
	GA	58.1		60.4	
	AA	53.2		60.6	
intermediate	GG	61.7	0.244	49.5	0.238
	GA	63.1		50.6	
	AA	63.4		46.6	
high	GG	61.6	0.683	45.1	0.208
	GA	61.4		47.0	
	AA	66.6		52.7	

[+]one case only.
[#]no calculation since all cases censored.
[*]surrogate definitions of intrinsic subtypes determined by immunohistochemistry.

Supplementary Table 10: Correlation of *MDMX* SNP34091-allele status with clinicopathogical parameters

variables	genotype	OR	95% confidence interval	p^*
age at diagnosis	A/A;A/C	1,000		
(< 40 yrs# vs. ≥ 40 yrs)	C/C	1,672	0,762–3,668	0,2
age at diagnosis	A/A;A/C	1,000		
(< 55 yrs# vs. ≥ 55 yrs)	C/C	0,954	0,712–1,277	0,751
primary tumor (T)	A/A;A/C	1,000		
(T1# vs. T2-T4)	**C/C**	**1,412**	**1,071–1,86**	**0,014**
nodal status (N)	A/A;A/C	1,000		
(N0# vs. N1-N3)	C/C	1,101	0,83–1,46	0,503
grading	A/A;A/C	1,000		
(G1/2# vs. G3)	C/C	0,709	0,46–1,092	0,119
histological subtype	A/A;A/C	1,000		
(lobular# vs. NST)	C/C	1,168	0,794–1,718	0,429
lymph vessel invasion	A/A;A/C	1,000		
(V0# vs. V1)	C/C	0,918	0,674–1,25	0,589
ER-status < 60 yrs.	A/A	1,000		
(ER-pos. # vs. ER-neg)	**A/C+C/C**	**0,546**	**0,304–0,978**	**0,042**
PR-status	A/A;A/C	1,000		
(PR-pos. # vs. PR-neg)	**C/C**	**1,452**	**1,073–1,964**	**0,016**
HER2-status	A/A;A/C	1,000		
(HER2-pos. # vs. HER2-neg.)	C/C	0,815	0,5461,217	0,317

OR: odds ratio; #reference group; *p-value from binary-logistic regressions.

Supplementary Table 11: Kaplan-Meier analysis of age-at-diagnosis and event-free survival for *MDMX* SNP34091

Variables	Genotype	age-at-diagnosis		event-free survival	
		years	*P*	*months*	*P*
**all patients**	AA	62.2	0.222	77.1	0.219
	AC	62.2		73.4	
	CC	64.6		67.3	
***TP53* mutations**					
wild-type	AA	62.7	0.809	78.3	0.766
	AC	62.3		74.5	
	CC	61.6		69.1	
mutated	AA	60.3	0.310	--[#]	
	AC	64.7		--	
	CC	50.3		--	
**ER-status**					
negative	AA	59.3	0.633	64.6	0.924
	AC	64.4		63.9	
	CC	61.7		59.7	
positive	AA	62.7	0.219	78.7	0.131
	AC	61.8		74.7	
	CC	65.1		68.2	
**HER2-status**					
negative	AA	62.7	0.245	77.6	0.268
	AC	62.1		74.0	
	CC	64.2		67.8	
positive	AA	58.2	0.391	70.6	0.317
	AC	60.9		64.8	
	CC	71.7		50.8	
**intrinsic subtypes**[*]					
Luminal A-like	AA	61.5	0.478	80.5	0.252
	AC	61.8		74.8	
	CC	64.2		73.2	
Luminal B-like	AA	63.1	0.063	74.0	0.001
	AC	60.7		70.6	
	CC	67.0		54.8	
HER2-positive (non-luminal)	AA	60.6	0.701	67.8	--
	AC	60.4		59.9	
	CC	76.0[*]		--	
triple negative (ductal)	AA	59.4	0.726	60.5	0.805
	AC	66.6		63.9	
	CC	59.7		64.8	
**St. Gallen risk groups**					
low	AA	58.3	0.162	52.1	0.747
	AC	59.6		54.1	
	CC	51.7		64.3	
intermediate	AA	62.5	0.224	50.8	0.174
	AC	62.3		48.4	
	CC	64.3		51.8	
high	AA	61.4	0.352	45.6	0.349
	AC	60.9		48.1	
	CC	71.7		46.3	

[#]no calculation since all cases censored.
[*]surrogate definitions of intrinsic subtypes determined by immunohistochemistry.
